# Deontologists are not always trusted over utilitarians: revisiting inferences of trustworthiness from moral judgments

**DOI:** 10.1038/s41598-023-27943-3

**Published:** 2023-01-30

**Authors:** Dries H. Bostyn, Subramanya Prasad Chandrashekar, Arne Roets

**Affiliations:** 1 Department of Developmental, Personality, and Social Psychology, Ghent University, Henri Dunantlaan 2, 9000 Ghent, Belgium; 2 Department of Psychology, Norwegian University of Science and Technology (NTNU), Edvard Bulls veg 1, 7049 Trondheim, Norway

**Keywords:** Human behaviour, Psychology

## Abstract

**Abstract:**

Recent research has looked at how people infer the moral character of others based on how they resolve sacrificial moral dilemmas. Previous studies provide consistent evidence for the prediction that those who endorse outcome-maximizing, utilitarian judgments are disfavored in social dilemmas and are seen as less trustworthy in comparison to those who support harm-rejecting deontological judgments. However, research investigating this topic has studied a limited set of sacrificial dilemmas and did not test to what extent these effects might be moderated by specific features of the situation described in the sacrificial dilemma (for instance, whether the dilemma involves mortal or non-mortal harm). In the current manuscript, we assessed the robustness of previous findings by exploring how trust inference of utilitarian and deontological decision makers is moderated by five different contextual factors (such as whether the sacrificial harm is accomplished by an action or inaction), as well as by participants’ own moral preferences. While we find some evidence that trust perceptions of others are moderated by dilemma features, we find a much stronger effect of participants’ own moral preference: deontologists favored other deontologists and utilitarians favored utilitarians.

**Protocol registration:**

The stage 1 protocol for this Registered Report was accepted in principle on 21 September 2022. The protocol, as accepted by the journal, can be found at: 10.6084/m9.figshare.21325953.

## Introduction

Our ability to quickly and accurately infer the moral character of others is critical for successfully navigating the social world around us. Research on social perception supports the view that one of the first aspects we notice about others is whether they intend to be harmful or helpful^1,2^. The moral values that people profess to have and the judgments they make, reveal crucial information about someone’s character and are invaluable for deciding who to trust^1,3,4^. As can be expected, we tend to like and trust others who share our values more than those who do not^5,6^. However, prior research has indicated that some displays of particular moral principles seem to affect our perception of others’ trustworthiness, regardless of whether they correspond with our own principles.

In a series of seminal studies, Everett et al.^7^ confronted participants with the choices other people had made on hypothetical sacrificial moral dilemmas and investigated to what extent this influenced how trustworthy these people were perceived to be. Sacrificial dilemmas revolve around a trade-off between different moral values, typically pitching a moral concern not to harm others against a desire to minimize overall harm^8,9^. Imagine, for instance, a scenario in which a runaway trolley train is about to hit and kill five unsuspecting workmen. The only way to save the group of five is to divert the train to a second track where it will hit and kill a single workman instead. Everett et al. uncovered that participants tended to trust those that favored not actively harming others, i.e., not sacrificing the single man at the expense of the group, over those that did interfere to minimize overall harm; a result that has been replicated in several other studies^10–14^. In reference to philosophical theory, decisions to minimize overall harm are often labeled as “utilitarian” or “consequentialist”, as these necessitate a focus on the outcome of the scenario and revolve around minimizing overall harm. Conversely, decisions to reject doing any harm to others are labeled “deontological” as they are broadly consistent with absolute condemnations of harm, a hallmark of deontological approaches to ethics. While the appropriateness of these labels is actively debated^10,15,16^, the findings of Everett et al.^7^ are often summarized as implying that people trust deontologists more than utilitarians.

As is so often the case, summaries tend to gloss over crucial nuances. In the initial set of studies that Everett et al.^7^ presented, they did not find that utilitarians were distrusted in *all* dilemma contexts. In fact, in some studies, they found that utilitarians were trusted over deontologists. More specifically, when participants were confronted with a sacrificial dilemma about an injured soldier who was bound to be captured by enemy forces and who requested to be killed to escape torture, those favoring mercy killing (the utilitarian option) were trusted over those who favored leaving the soldier behind (the deontological option).

**Figure 1:**
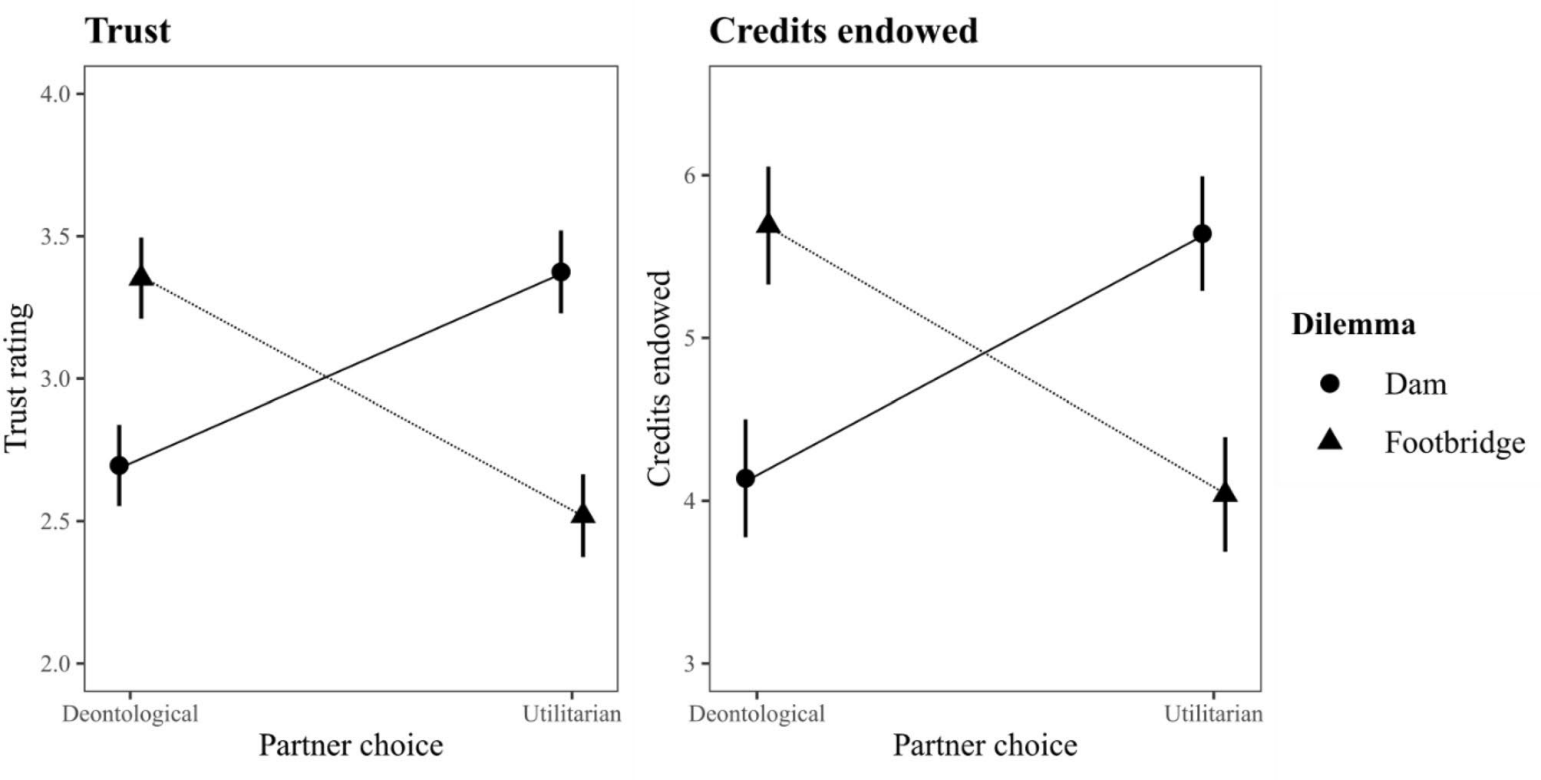
Inferred trust and cooperation in the pilot study as a function of partner judgment and dilemma type (n = 272).

Importantly, contexts in which utilitarians are trusted over deontologists are not limited to specific scenarios where the sacrificial victim consents to the sacrificial harm. In the supplementary materials, we report a pilot study in which participants were asked how much they trusted and were willing to cooperate with others that had responded with a utilitarian or deontological response to a moral dilemma. When participants read about someone responding to the footbridge dilemma, a variant of the trolley dilemma described above, where the sacrificial harm necessitates actively pushing a man to his death to save five others, people favored those disavowing the sacrificial harm over those condoning it; a replication of the effect found throughout the literature. However, when confronted with a dam dilemma where a smaller town needs to be flooded to save a larger town from flooding, the pattern is reversed (see Fig. 1). Accordingly, while many studies have confirmed that, by and large, we tend to trust deontologists over utilitarians, this effect does appear to be dependent on the specific dilemma people are confronted with.

The relevance of research that delves further into which features of moral dilemma decisions impact trust perceptions has recently been highlighted by the COVID-19 pandemic. People's trust in their government and their leaders is essential for a constructive response to crises, as governmental trust has proven to be a strong predictor of citizen compliance with public health policy measures. The importance of public trust appears to be especially salient when leaders seek a change in behavior that comes with personal costs^17–21^ (e.g., self-isolation, social distancing). Many of the choices that governments were required to make on pandemic measures involved sacrificial trade-offs. For example, during the initial rollout of vaccines, governments were faced with the question of which groups to vaccinate first as rollout policies can prioritize different parts of the population. Generally speaking, governments have a strong incentive to favor utilitarian decisions as these focus on minimizing total harm. However, if such utilitarian decisions lower trust in government, they might ultimately be less beneficial than they appear to be, and come with a risk of backfiring. Examining when utilitarian decisions have and do not have negative consequences on trust perceptions hence will not only provide new insights into our moral minds but has policy relevance as well.

In the current registered report, we report the results of a study in which we empirically investigate how sensitive trust inference is to a variety of dilemma factors. We investigated not just to what extent trust inference is impacted by whether a decision is deontological or utilitarian, but examined whether (and to what extent) differences in trust inference are moderated by (1) whether the sacrificial harm necessitates an action or an inaction, (2) the degree of the consequential benefit (e.g. saving three lives vs. saving ten lives), (3) whether the sacrificial harm involves mortal harms (deadly vs. non-deadly harm), (4) whether the sacrificial harm is instrumental in obtaining the consequential benefit (instrumental vs. non-instrumental), and finally, (5) whether the decision-maker is causally responsible for the dilemma situation (responsible vs. not casually responsible). To delineate the impact of each dilemma feature, we manipulated all features within one single dilemma context and explored how each of them impacts trust perceptions. Additionally, we investigated to what extent trust perceptions were moderated by participants’ own moral preferences. In what follows, we discuss each of the five dilemma features in more detail.

### Dilemma features beyond the deontological vs. utilitarian contrast

#### Action vs. inaction

In a typical sacrificial dilemma, participants are asked to choose between two options: harming an individual to save a greater number of people (the utilitarian decision) or refusing to commit such harm at the expense of the larger group (the deontological decision). On nearly all sacrificial dilemmas used throughout the literature, the utilitarian decision corresponds with an active decision (e.g., *pushing* a man in front of the trolley) whereas the deontological decision corresponds with inaction (e.g., *allowing* the trolley to continue down its current path). Recently, some researchers have developed novel research paradigms that are explicitly aimed at controlling for this potential confound (see, for instance^22^ or^23^). This entails that the type of decision (utilitarian vs. deontological) tends to be confounded by whether the decided course involves an action or not. This is not a trivial confound, as prior research has established that active harms are considered more morally inappropriate than similar harms resulting from inaction^22,24–28^. Importantly, none of the existing studies investigating how moral decisions impact trust perceptions has controlled for this confound. It is therefore unclear to what extent differences in trust perceptions are caused by the utilitarian or the active nature of the choice that results in harm.

#### Degree of consequential benefit

Sacrificial harms differ in the extent to which they cause an overall benefit. Unsurprisingly, research has demonstrated that utilitarian decisions are more likely to be approved when the benefit is larger than when it is smaller^29^. It is reasonable to suppose that the extent to which people trust (or distrust) utilitarians is also impacted by the size of the consequentialist benefit. However, whether the resulting effect is either large or small is an open research question. Could a large utilitarian benefit be sufficient to shelter utilitarians from distrust?

#### The mortality of harm caused

Sacrificial dilemmas typically describe trade-offs involving lives. However, there is no a priori reason why sacrificial dilemmas should only involve mortal harm and indeed, some studies have explored sacrificial dilemmas that revolve around non-mortal harms (e.g.^30^). While these studies have mostly uncovered that people tend to approach such dilemmas similarly to how they approach mortal harm dilemmas^31,32^, this distinction may nevertheless impact trust perceptions. Deciding in favor of active mortal harm is fundamentally different from deciding in favor of active non-mortal harm; if for no other reason than that the former is simply more definitive than the latter. A victim of non-mortal sacrificial harm can be compensated after the fact, the same is not true for a victim of mortal harm. As such, people might be less suspicious of utilitarian others when they endorse committing non-mortal compared to mortal sacrificial harm.

#### Instrumentality of the harm

In some dilemmas, sacrificial harm is an accidental side-effect of pursuing the consequential benefit. For instance, in the original trolley dilemma, the trolley train is diverted to a second track. While it is unfortunate that this causes another man -who happens to be on that track- to be run over, the sacrifice is not instrumental in saving the group of five. In contrast, in the footbridge variant of the dilemma, a large man needs to be pushed in front of the trolley to save the group of five. In the latter dilemma, the sacrificial harm is causally responsible for obtaining the consequential benefit. Prior research has demonstrated that approval of sacrificial harm tends to be lower when the sacrificial harm is instrumental rather than accidental (see for instance^26,33–35^). Accordingly, it seems reasonable to suppose that people might be more suspicious of utilitarian decisions whenever sacrificial harm is instrumental.

#### Whether the protagonist caused the dilemma situation

In many sacrificial dilemmas, the protagonist in the dilemma scenario is an outsider who is forced to make a difficult decision about a situation for which they are not responsible (see, the footbridge dilemma described above). However, in real life, many of the difficult decisions that people are required to make are direct (although often unintended) consequences of prior decisions they have made. Therefore, when someone has created a dilemma-like situation (for instance, by accident), they are more responsible for resolving that situation than when they are a bystander drawn into the problem by chance. When someone has caused a dilemma situation, they might be expected to want to ensure that the harm caused by their prior choices is as limited as possible. This might impact how utilitarian decisions are perceived as well.

### In summary

People infer how trustworthy others are from the moral decisions they make. Prior research has established that, by and large, people that favor deontological judgments are trusted over those that favor utilitarian judgments. In the current manuscript, we aim to revisit and build on the existing literature by investigating to what extent specific features of the dilemma context and participants’ own moral judgments moderate this effect.

### Open science declaration

We report how we determine our sample size, all data exclusions (if any), all manipulations, and all measures in the study. All materials, data, and statistical code are available on https://osf.io/xayu7. The preregistration for this study is available at 10.17605/OSF.IO/BY23C. No data was collected before the acceptance in principle date. Please note that while we did preregister analysis plans, we did not preregister specific hypotheses.

### Ethical approval

The study has been approved by the Ethical Guidance committee of the Faculty of Psychology and Educational Sciences at Ghent University (2022-41 Bostyn). All the study procedures and methods noted in the MS are in accordance with the relevant guidelines and regulations. We invited participants older than 18 years. Informed consent was obtained from all participants of the study.

## Study

### Design

#### Participants and sample size

We collected a sample of 1400 North-American participants on Amazon Mechanical Turk through the CloudResearch platform. Participants using suspicious geocode locations, and those with duplicate IP addresses were blocked from participation. Additionally, we sampled participants from CloudResearch’s list of approved participants and only used Mturk survey takers with a minimum HIT approval rate of 90%. Participants were paid US$1.00 to complete the survey and were able to earn an additional bonus based on their responses (see infra).

Sample size was based on a power analysis using the data gathered in the pilot. We were mainly interested in either main effects of dilemma factors, or in interaction effects of these dilemma features with participants’ own moral preference (i.e., whether they responded in a utilitarian or deontological fashion to the dilemma). Given that interaction effects are typically less powerful than main effects, we based our power analysis on the power for finding interactions. Using the *simr* package^36^ in R (Version 1.06) we found that the current design would have 80% power for interaction effects that are 1/4th the size of the effect we found in the pilot and 97% power for interaction effects that are 1/3rd that size. An R script to repeat this power analysis is available at https://osf.io/xayu7.

As per our preregistration, participants were excluded from the main analyses if they met at least one of the following criteria: (1) they indicated a low proficiency in English (self-report < 5, on a 1–7 scale); (2) self-reported not being serious about filling in the survey (self-report < 4, on a 1–5 scale); (3) completed the survey too quickly (within 1 min); or (4) failed any of the two comprehension checks. Based on our pilot study (that used the same exclusion criteria, we expected a drop-out rate of 10%. However, 470 participants failed to meet these criteria, mainly, because 425 participants failed the first comprehension check. In hindsight, this comprehension check might have been formulated confusingly. We did not preregister any conditions for additional data collection, so we refrained from collecting additional data. While the high rate of exclusions is unfortunate, none of our conclusions depend on whether the sample is analyzed with or without exclusions. For nearly all reported analyses, results are qualitatively the same whether we use the full sample or the sample with exclusions. We explicitly note any differences between analyses conducted on the reduced sample versus those on the full sample in the main text. All results are available through the supplementary materials.

Our final sample contained 930 participants with an average age of 41.88, of which 483 self-identified as “male”, 430 self-identified as “female”, 8 identified as “other” and 9 participants preferred not to disclose their gender identity. A total of 722 participants identified as white, 53 as Hispanic or Latino, 65 as Black or African American, 4 as Native American or American Indian, 74 as Asian American or Pacific Islander and 12 as Other.

#### Procedure

First, participants were asked to provide their informed consent. Subsequently, participants were asked to play a series of nine trust games. The first of these trust games was an *uninformed* trust game, aimed at measuring participants' baseline levels of trust and cooperation. The following eight trust games were *informed* trust games in which participants were provided with some information about their partner in the trust game: their decision on a sacrificial dilemma. All the dilemmas displayed to participants were a variation of the same core dilemma in which the five contextual factors have been independently manipulated. The informed trust games served a dual purpose: (a) they were used to measure trust in and cooperation with people preferring deontological or utilitarian judgments, and (b) they polled participants for their opinion of the sacrificial dilemma they were presented with as a measure of their own moral preferences.

Once all trust games were completed, participants responded to a series of questions aimed at probing data quality. Participants were asked how serious they were when completing the survey, whether they had seen the materials used in this study before, what they thought the purpose of the study was, to rate their understanding of the English used in the study, whether they had any comments about how we could better run these types of studies and if they were satisfied with their payment. Finally, participants were asked to report their gender, age, country of birth, subjective SES, income, religion and religiosity, ethnicity, and political orientation.

### Materials

#### Trust games

Participants were invited to play a series of nine trust games. Before playing their first trust game, participants were explained how trust games work. More specifically, participants were explained that each trust game has two players: a trustor and a trustee. Participants were told that the trustor would receive 10 credits and that they were free to decide how many of these credits to give to the trustee. They were told that all credits given to the trustee would be tripled and that afterward, the trustee was free to decide how many of the credits they wished to return. Trustors could thus maximize their earnings by endowing the trustee with a large number of credits but in doing so they risked losing out on earnings if they decided to endow an untrustworthy trustee with their credits. Participants were told that they would play a series of these games and that they would be matched with a different partner on each game. Participants were told they would not be playing these games live but that their responses would be randomly matched to those of other participants after the study was completed. We told participants that they would play games as either the trustor or the trustee but in reality, all participants played all games from the perspective of the trustor.

After receiving instructions on the trust game, participants were asked two comprehension questions. First, they were asked to imagine a situation where the trustor transferred 5 credits to the trustee and asked how many credits (if any) the trustee should return if the trustee wants to maximize their earnings. Participants were asked to respond using a multiple-choice format and were presented with four choice options: returning 0, 5, 10 or 15 credits. Subsequently, they were asked how many credits a trustee would receive if a trustor endows them with 10 credits and had to select the correct response from the options: 10, 20, 30, or 40 credits.

After completing the comprehension checks participants started playing the trust games. First, they played an *uninformed* trust game. Participants were told they were matched up with a random other participant about whom they received no information. They were asked to rate how much they trusted this random other person using a five-point Likert scale spanning from (1) *Not at all* to (5) *Completely.* They were also asked how many credits they wanted to endow this random other person with, using a sliding scale spanning from 0 to 10 credits.

Subsequently, they played an additional eight *informed* trust games. In each of these trust games, participants were shown a sacrificial dilemma and were told what their supposed partner's response was to this sacrificial dilemma. Participants were presented with four partners that responded in a deontological fashion and four partners that responded in a utilitarian fashion. In each of these games, participants were asked how much they trusted their partner in the trust game and how many credits they wanted to endow them with using the same scales as above. Subsequently, they were polled about how they would respond to the dilemma themselves. We asked participants whether they themselves would opt for the sacrificial harm in a binary manner (Yes or No) and to rate how morally appropriate they thought both options in the dilemma were using a seven-point scale going from (1) Absolutely Inappropriate to (7) Absolutely Appropriate.

The dilemmas displayed to participants in this phase of the experiment were variations on the same base dilemma. On each trust game, one variation was randomly selected. These dilemmas differed across the five factors of interest based on (1) whether the sacrificial harm necessitates an action or an inaction, (2) the degree of the consequential benefit (saving five lives vs. two lives), (3) whether the sacrificial harm revolves around mortal harms (deadly vs. non-deadly harm), (4) instrumentality of the sacrificial harm (yes vs. no), (5) whether the decision-maker is causally responsible for the dilemma situation (responsible vs. not casually responsible). Each participant was paired up with eight different variations, and paired up with four deontological and four utilitarian partners.

The dilemmas provided to participants were constructed as per Table 1.

**Table 1:** Overview of the dilemma and the included dilemma factors.

Dilemma text	Manipulation
*Imagine the following scenario. You are a doctor working at a military hospital near the frontlines of an ongoing war*	*–*
***[A bomb has just been dropped on the hospital/while repairing crucial infrastructure, you accidentally caused an explosion]***	Whether the decision-maker is causally responsible
*The explosion has ruptured a gas-line and damaged several ventilation systems*	*–*
*Gas is leaking into a room that contains ****[three/ten]**** patients recovering from a severe injury*	Size of the consequential benefit
***[The gas is not life-threatening but will cause the patients to be in excruciating pain for at least one week./The gas is lethal and will cause the death of these patients]***	Mortality of harms
*The patients cannot be evacuated but there is a ventilation system installed in the room*	*–*
***[You can activate an alarm by pushing a button/An automatic alarm system has been activated which you can shut down by pushing a button.]***	Action/inaction
***[The alarm will trigger the ventilation system. The ventilation system will vent the gas into a nearby room that is guarded by a single soldier/The alarm will cause a soldier in a nearby guard post to activate the ventilation. You know the ventilation system will vent the gas into the room where the ventilation system is activated. You also know that the soldier is unaware of this.]***	Instrumentality of harm
*If the ventilation system is activated, the soldier will be incapacitated by the gas before they are able to evacuate the room and will suffer the consequences of the gas leak instead. Is it morally appropriate to *[*…*]	

Finally, to ensure that participants took these trust games seriously, they were told that they could earn a bonus spanning from US$0.00 to US$0.20 depending on how they responded to the dilemmas. To determine these bonuses, we used their responses to the uninformed trust game and paired all participants with a trustee that returned 2/3rd of the credits that were endowed to them and had each credit correspond to a dollar value of US$0.01.

## Results

All analyses were conducted in R using the *lme4* R package^37^.

### Replication analysis

We first tested the replicability of the effect that has been established in the literature, i.e., that participants report higher trust in, and are more likely to cooperate with deontological partners compared to utilitarian partners^7,10–14^. To test this hypothesis, we conducted separate analyses for the two dependent measures (trust and the number of credits endowed). We ran four linear mixed-effects models, one for each of the two dependent measures, using either the full sample or the sample with exclusions. We included their partner’s choice (Utilitarian vs. Deontological) as a predictor. Additionally, to control for participants’ own moral preferences, the binary decision they made on each dilemma was included as a control variable, as was the interaction of this variable with partner choice. All models included Participant ID as a random intercept. We used dummy coding for all categorical variables. We also repeated all analyses while controlling for participants’ baseline levels of trust (for the analysis with trust as a dependent measure) or their baseline levels of cooperation (for the analysis with credits as a dependent measure).

**Figure 2:**
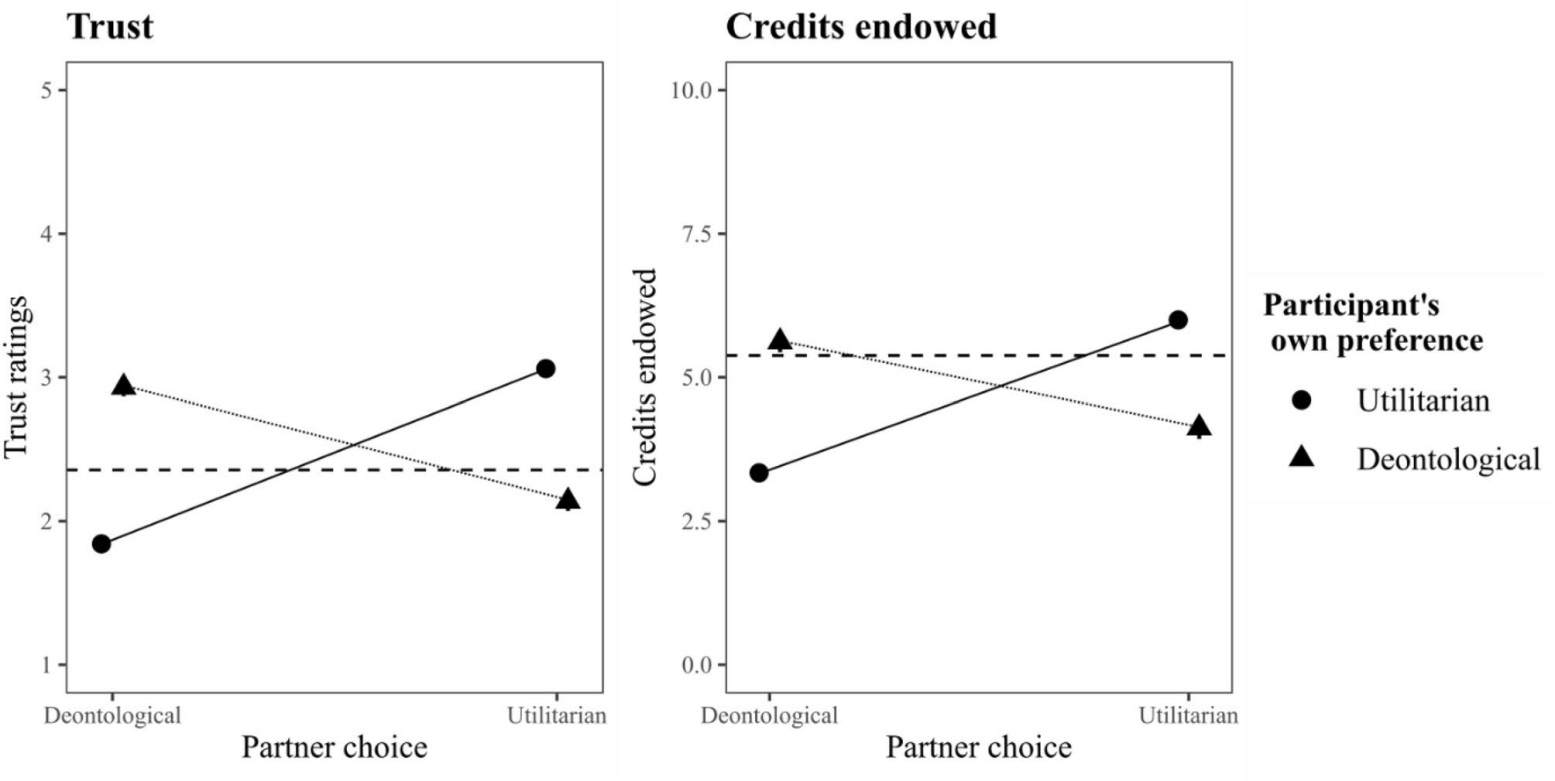
Inferred trust and cooperation in the main study as a function of partner judgment and participants’ own moral preference. Horizontal line designates average trust in or credits endowed to an unknown partner. *Note.* 95% Confidence intervals have been drawn but are not easily visible due to their small size.

Regression tables for the analyses with the baseline controls are available in Table 2. Importantly, results are qualitatively the same when we do not include these control variables. For ease of interpretation, the results are summarized in Fig. 2.

**Table 2:** Fixed effects for the replication analyses. Categorical variables were dummy coded. Top line displays the result using the sample with preregistered exclusions. Bottom line displays results using the full sample.

Trust ~	$\hat{b}$	*se*	*df*	*t*	*p*
Intercept	0.71	0.05	1020	15.20	< 0.001
	0.72	0.03	1523	18.38	< 0.001
Partner Choice: Utilitarian	1.22	0.02	6549	68.77	< 0.001
	1.13	0.02	9868	73.62	< 0.001
Own: Deontological	1.09	0.03	7431	33.57	< 0.001
	0.98	0.03	11,160	37.20	< 0.001
Base trust	0.48	0.02	926	27.10	< 0.001
	0.51	0.01	1397	35.79	< 0.001
Partner Choice × Own Preference	− 2.01	0.04	6702	− 48.98	< 0.001
	− 1.76	0.03	10,121	− 52.01	< 0.001

Credits ~	$\hat{b}$	*se*	*df*	*t*	*p*
Intercept	− 0.62	0.10	1082	− 6.12	< 0.001
	− 0.43	0.09	1609	− 4.99	< 0.001
Partner Choice: Utilitarian	2.66	0.05	6539	55.14	< 0.001
	2.45	0.04	9850	62.39	< 0.001
Own: Deontological	2.27	0.09	7380	25.46	< 0.001
	2.04	0.07	11,038	29.64	< 0.001
Base Credits	0.74	0.01	925	49.70	< 0.001
	0.73	0.01	1394	56.94	< 0.001
Partner Choice × Own Preference	− 4.15	0.11	6660	− 37.13	< 0.001
	− 3.68	0.09	10,043	− 42.26	< 0.001

Coding of the dummy variables: (1) Partner choice: 1 = Utilitarian, 0 = Deontological; (2) Own preference (i.e., participant's own preference): 0 = Utilitarian, 1 = Deontological.

As mentioned previously, the existing literature has claimed that people trust deontologists over utilitarians. In contrast, we find that participants’ trust in utilitarians and deontologists greatly depends on their own moral preference. As Fig. 2 demonstrates, in comparison to their trust in an unknown partner, participants favoring utilitarian judgment displayed increased trust in utilitarians and decreased trust in deontologists. In contrast, participants favoring deontological judgment showed more trust in deontologists and distrusted utilitarians. Put simply, participants favored those with the same moral preference and disfavored those with a differing perspective. Because the majority of participants favored a utilitarian response to the dilemma(s) we used, we also find that, overall, utilitarians were trusted over deontologists, ${t}_{trust}(6509)$ = 44.50, *p* < 0.001; ${t}_{credits}(6509)$ = 38.86, *p* < 0.001.

### Main analysis

Subsequently, we explored the effect of the various dilemma features on participants’ trust in others and their level of cooperation. We analyzed the influence of each feature separately. For each of these, we ran four mixed-effects regression models. We ran analyses both using the full sample and the sample with preregistered exclusions and with trust ratings or endowed credits as the dependent measure. In all models, we included the dilemma feature as a factor, participants’ own moral preference and their partner’s choice, as well as all interactions between these variables as predictors. Additionally, we included participants’ baseline levels of trust or cooperation as control variables. We used dummy coding for all categorical variables. Participant IDs were included as random intercepts.

While we did not preregister such analyses, the overall impact of a feature is perhaps best assessed by conducting *χ*^2^ model comparison tests. By comparing a model that includes a feature as a predictor (and all its interactions) to a model without said feature we can test the overall, combined impact of the main effect and all interaction effects of each feature. Such tests suggested that some features did have an effect whereas others did not. For the sake of brevity, the mixed effects regression tables for features that did not have an effect are available through the supplementary materials rather than in the main text.

Two features did not appear to have an effect. Both instrumentality and whether the decision-maker had caused the dilemma situation failed to increase model fit no matter which sample or dependent variable we analyzed (all *χ*^2^(4) < 8.38, all *p* > 0.079). Additionally, one feature had an inconsistent impact depending on which sample we analyzed. While we found no effect of the action-inaction distinction when using the preregistered exclusions (both *χ*^2^(4) < 9.32, both *p* > 0.054), we did find an effect when analyzing the data using the full sample (both *χ*^2^(4) > 16.83, both *p* < 0.002). To the extent that the effects of this feature should be interpreted, Table 3 suggests that participants trusted deontologists slightly less when deontological judgment required an action.

**Table 3:** Effects of the action-feature. Categorical variables were dummy coded. Top line displays the result using the sample with preregistered exclusions. Bottom line displays results using the full sample.

Trust ~	$\hat{b}$	*se*	*df*	*t*	*p*
Intercept	0.73	0.05	1213	15.09	< 0.001
	0.78	0.04	1826	18.80	< 0.001
Partner Choice: Utilitarian	1.22	0.03	6693	45.87	< 0.001
	1.10	0.03	10,087	48.34	< 0.001
Own Preference: Deontological	1.07	0.05	7373	26.27	< 0.001
	0.97	0.04	11,021	28.68	< 0.001
Inaction	−0.05	0.03	6874	− 1.87	0.061
	− 0.10	0.02	10,340	− 4.17	< 0.001
Base trust	0.48	0.02	926	27.12	< 0.001
	0.51	0.01	1398	35.80	< 0.001
Partner Choice × Own Preference	− 2.04	0.05	6741	− 38.52	< 0.001
	− 1.84	0.05	10,170	− 40.60	< 0.001
Partner Choice × Inaction	− 0.01	0.04	6824	− 0.26	0.798
	0.05	0.03	10,283	1.65	0.100
Own: Deo × Inaction	0.02	0.06	6999	0.29	0.774
	− 0.02	0.05	10,533	− 0.33	0.743
Partner Choice × Own × Inaction	0.07	0.09	6803	0.86	0.393
	0.20	0.07	10,231	2.87	0.004

Credits ~	$\hat{b}$	*se*	*df*	*t*	*p*
Intercept	− 0.57	0.11	1419	− 5.29	< 0.001
	− 0.33	0.09	2045	− 3.55	< 0.001
Partner Choice: Utilitarian	2.65	0.07	6652	36.49	< 0.001
	2.37	0.06	10,016	40.50	< 0.001
Own Preference: Deontological	2.24	0.11	7286	20.03	< 0.001
	2.02	0.09	10,849	22.87	< 0.001
Inaction	− 0.08	0.07	6801	− 1.05	0.293
	− 0.20	0.06	10,216	− 3.29	< 0.001
Base trust	0.74	0.01	925	49.70	< 0.001
	0.73	0.01	1394	56.96	< 0.001
Partner Choice × Own Preference	− 4.12	0.14	6690	-28.68	< 0.001
	− 3.74	0.12	10,080	-32.13	< 0.001
Partner Choice × Inaction	0.01	0.10	6758	0.10	0.918
	0.16	0.08	10,169	1.89	0.059
Own: Deo × Inaction	0.03	0.17	6897	0.21	0.836
	− 0.03	0.13	10,378	− 0.22	0.826
Partner Choice × Own × Inaction	− 0.04	0.24	6740	− 0.18	0.854
	0.20	0.18	10,127	1.10	0.273

Coding of the dummy variables: (1) Partner choice: 1 = Utilitarian, 0 = Deontological; (2) Own preference (i.e., participant's own preference): 0 = Utilitarian, 1 = Deontological; (3) Action frame: 1 = Inaction, 0 = Action.

The remaining two features did have a consistent effect. Whether the dilemma included mortal harm and whether the size of the sacrificial benefit was either large or small impacted trust perception and cooperation in both samples (all *χ*^2^(4) < 11.53, all *p* < 0.021). As Table 4 demonstrates, participants trusted deontological partners less when dilemmas involved mortal harm and trusted utilitarians more. Similarly, Table 5 shows that participants trusted deontologists more when the sacrificial benefit was small and trusted utilitarians more when the sacrificial benefit was large.

**Table 4:** Effects of the mortality-feature. Categorical variables were dummy coded. Top line displays the result using the sample with preregistered exclusions. Bottom line displays results using the full sample.

Trust ~	$\hat{b}$	*se*	*df*	*t*	*p*
Intercept	0.76	0.05	1214	15.60	< 0.001
	0.77	0.04	1810	18.71	< 0.001
Partner choice: Uti	1.16	0.03	6670	44.43	< 0.001
	1.08	0.02	10,099	48.09	< 0.001
Own: Deo	1.06	0.04	7275	24.56	< 0.001
	0.95	0.03	10,930	27.33	< 0.001
Mortality: yes	− 0.10	0.03	6799	− 3.62	< 0.001
	− 0.09	0.02	10,261	− 3.87	< 0.001
Base trust	0.48	0.02	926	27.11	< 0.001
	0.51	0.01	1397	35.81	< 0.001
Partner choice: Uti × Own: Deo	− 1.96	0.06	6754	− 33.81	< 0.001
	− 1.71	0.05	10,180	− 36.48	< 0.001
Partner Choice: Uti × Mortality	0.10	0.04	6830	2.73	0.006
	0.09	0.03	10,298	2.65	< 0.001
Own: Deo × Mortality	0.04	0.06	6875	0.76	0.450
	0.05	0.04	10,361	1.12	0.262
Partner Choice × Own × Mortal	− 0.09	0.08	6817	− 1.13	0.260
	− 0.10	0.07	10,282	− 1.45	0.147

Credits ~	$\hat{b}$	*se*	*df*	*t*	*P*
Intercept	− 0.49	0.11	1415	− 4.52	< 0.001
	− 0.32	0.09	2033	− 3.45	< 0.001
Partner Choice: Uti	2.54	0.07	6658	35.63	< 0.001
	2.36	0.06	10,025	40.69	< 0.001
Own: Deo	2.20	0.12	7176	18.56	< 0.001
	1.96	0.09	10,758	27.73	< 0.001
Mortality	− 0.24	0.07	6737	− 3.27	0.001
	− 0.22	0.06	10,152	− 3.66	< 0.001
Base trust	0.74	0.01	925	49.70	< 0.001
	0.73	0.01	1394	56.85	< 0.001
Partner Choice: Uti × Own: Deo	− 4.11	0.16	6701	− 26.00	< 0.001
	− 3.60	0.12	10,087	− 29.87	< 0.001
Partner Choice: Uti × Mortality	0.21	0.10	6762	2.07	0.038
	0.18	0.08	10,180	2.24	0.025
Own: Deo × Mortality	0.12	0.16	6799	0.78	0.437
	0.13	0.12	10,230	1.07	0.284
Partner Choice × Own × Mortal	− 0.06	0.23	6751	− 0.28	0.777
	− 0.14	0.18	10,168	− 0.79	0.430

Coding of the dummy variables: (1) Partner choice: 1 = Utilitarian, 0 = Deontological; (2) Own preference (i.e., participant's own preference): 0 = Utilitarian, 1 = Deontological; (3) Mortal harm: 1 = Yes, 0 = No.

**Table 5:** Effects of the size-feature. Categorical variables were dummy coded. Top line displays the result using the sample with preregistered exclusions. Bottom line displays results using the full sample.

Trust ~	$\hat{b}$	*Se*	*df*	*t*	*p*
Intercept	0.65	0.05	1166	13.39	< 0.001
	0.66	0.04	10,087	54.42	< 0.001
Partner Choice: Uti	1.31	0.03	6695	51.44	< 0.001
	1.20	0.02	10,087	54.52	< 0.001
Own: Deo	1.19	0.05	7277	25.79	< 0.001
	1.08	0.04	10,903	28.79	< 0.001
Size: small	0.13	0.03	6775	5.07	< 0.001
	0.13	0.02	10,219	5.84	< 0.001
Base trust	0.48	0.02	926	27.07	< 0.001
	0.51	0.01	1397	35.80	< 0.001
Partner Choice: Uti × Own: Deo	− 2.09	0.06	6759	− 33.65	< 0.001
	− 1.81	0.05	10,231	− 35.69	< 0.001
Partner Choice: Uti × Small	− 0.18	0.04	6825	− 4.87	< 0.001
	− 0.14	0.03	10,282	− 4.33	< 0.001
Own: Deo × Small	− 0.19	0.06	6824	− 3.36	< 0.001
	− 0.19	0.05	10,295	− 3.95	< 0.001
Partner Choice × Own × Small	0.16	0.08	6814	1.93	0.054
	0.10	0.07	10,303	1.44	0.151

Credits ~	$\hat{b}$	*se*	*df*	*t*	*P*
Intercept	− 0.79	0.11	1333	− 7.38	< 0.001
	− 0.60	0.09	1959	− 6.59	< 0.001
Partner Choice: Uti	2.93	0.07	6653	42.32	< 0.001
	2.68	0.06	10,014	47.50	< 0.001
Own: Deo	2.52	0.13	7175	19.94	< 0.001
	2.30	0.10	10,729	23.68	< 0.001
Size: Small	0.36	0.07	6717	4.99	< 0.001
	0.35	0.06	10,115	5.92	< 0.001
Base trust	0.74	0.01	925	49.54	< 0.001
	0.73	0.01	1394	56.82	< 0.001
Partner Choice: Uti × Own: Deo	− 4.38	0.17	6704	− 25.92	< 0.001
	− 3.92	0.13	10,125	− 30.04	< 0.001
Partner Choice: Uti × Small	− 0.55	0.10	6757	− 5.45	< 0.001
	− 0.46	0.08	10,164	− 5.64	< 0.001
Own: Deo × Small	− 0.48	0.16	6756	− 3.05	0.002
	− 0.51	0.12	10,176	− 4.11	< 0.001
Partner Choice × Own × Small	0.48	0.23	6748	2.10	0.036
	0.47	0.18	10,182	2.65	0.008

Coding of the dummy variables: (1) Partner choice: 1 = Utilitarian, 0 = Deontological; (2) Own preference (i.e., participant's own preference): 0 = Utilitarian, 1 = Deontological; (3) Size: 1 = Small, 0 = Large.

**Figure 3:**
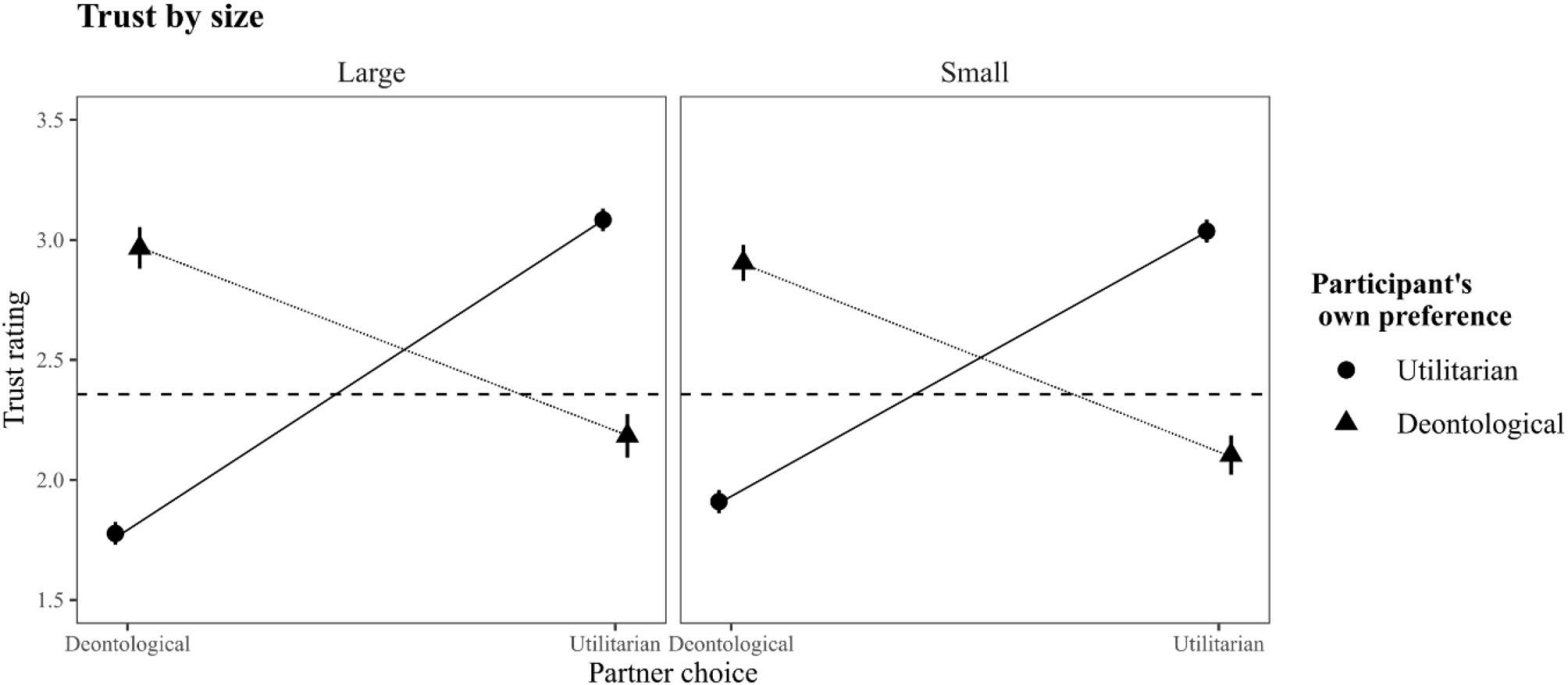
Inferred cooperation as a function of partner judgment, participants’ own moral preference, and whether the sacrificial benefit was either large or small. The horizontal line designates average trust in an unknown partner. *Note.* 95% Confidence intervals have been drawn but are not easily visible due to their small size.

While these findings demonstrate that the dilemma features we manipulated had an impact on trust and cooperation, these results should not be overstated. The effects we found of these features are rather small in comparison to the effect of participants’ own moral preference. This can be readily demonstrated visually. Figure 3 demonstrates the full 3-way interaction of participants' own moral preference, partner choice, and whether the sacrificial benefit was either large or small (i.e. the size feature) with trust ratings as the dependent. The size feature had the biggest impact of all features we tested, and yet as Fig. 3 demonstrates, this effect is much more subtle than the effect of participants’ own moral preference.

## Discussion

People judge other people based on the moral preferences those other people display. Within the context of sacrificial harm, prior research^10,11,38^ has suggested that people tend to trust those that disavow sacrificial harm (i.e., deontologists) over those that condone it (i.e., utilitarians). In the current study, we aimed to investigate to what extent this differential trust is moderated by features of the dilemma situation such as whether the sacrificial harm necessitates an action, whether it is instrumental in obtaining the sacrificial benefit, whether the decision-maker caused the dilemma situation, whether the dilemma involves mortal harms or whether the sacrificial benefit is large or small.

To eliminate the potential for any confounds, we manipulated each of these features within a single dilemma context and created 32 variations of the same dilemma. Interestingly, while we did find that some of these features influenced trust in utilitarian or deontological decision-makers, the effect of these features paled in comparison to the effect of participants’ own moral preferences. Utilitarians trusted utilitarian decision-makers more than they did anonymous decision-makers and trusted deontological decision-makers less. Similarly, deontologists favored deontologists and disfavored utilitarians. In the current study, most participants favored utilitarian judgment on the dilemmas we confronted them with, and as a result, we also found that overall, participants preferred utilitarians over deontologists.

These results seem to run against the findings that have been established in the literature^7,11,38^. Indeed, they even run against some of our own findings^10^. Yet our results are likely to appear more contradictory than they should. Ours is not the first study to uncover that in some dilemmas, utilitarians are trusted over deontologists. As we pointed out in our introduction: even the seminal work by Everett and colleagues^39^ reported such a finding when confronting people with the responses to a dilemma that involved a case of consensual euthanasia. In their work, Everett and colleagues explained that result by suggesting that people might be implicit consequentialists and suggested that explicit consent of the sacrificial victim was likely the key factor causing that result. Given that we found some effects of specific dilemma features in our work, it certainly seems possible that the consent of the sacrificial victim would moderate trust perceptions. However, the consensual euthanasia dilemma is also a dilemma on which a majority of people condone the sacrificial harm. As such, it might very well be the case that responses to that dilemma followed the pattern we uncovered in the current work: people trust those that display the same moral preference and when a majority of people prefer the utilitarian response, people will trust utilitarians over deontologists.

The results of the current work do not imply that dilemma features themselves are unimportant. While the features we manipulated only had a humble effect on trust perceptions, some of them still influenced trust perceptions above and beyond participants’ own moral preference. Furthermore, dilemma features have a direct impact on participants’ moral preference as well. The entire reason why people often prefer different responses to different dilemmas is that those dilemmas differ in some crucial way. Accordingly, the current results understate the impact of dilemma features on trust perceptions somewhat. By controlling for participants’ moral preferences, the effect of some features might have been subsumed in the effect of participants' own preference. An exploratory analysis suggests this might have occurred for the action feature. If we do not control for participants’ moral preference, the action feature does have an impact on trust, but because the action feature also impacted participants' own preference, the effect of action on trust disappears once we control for participants’ preference.

## Limitations

**Figure 4:**
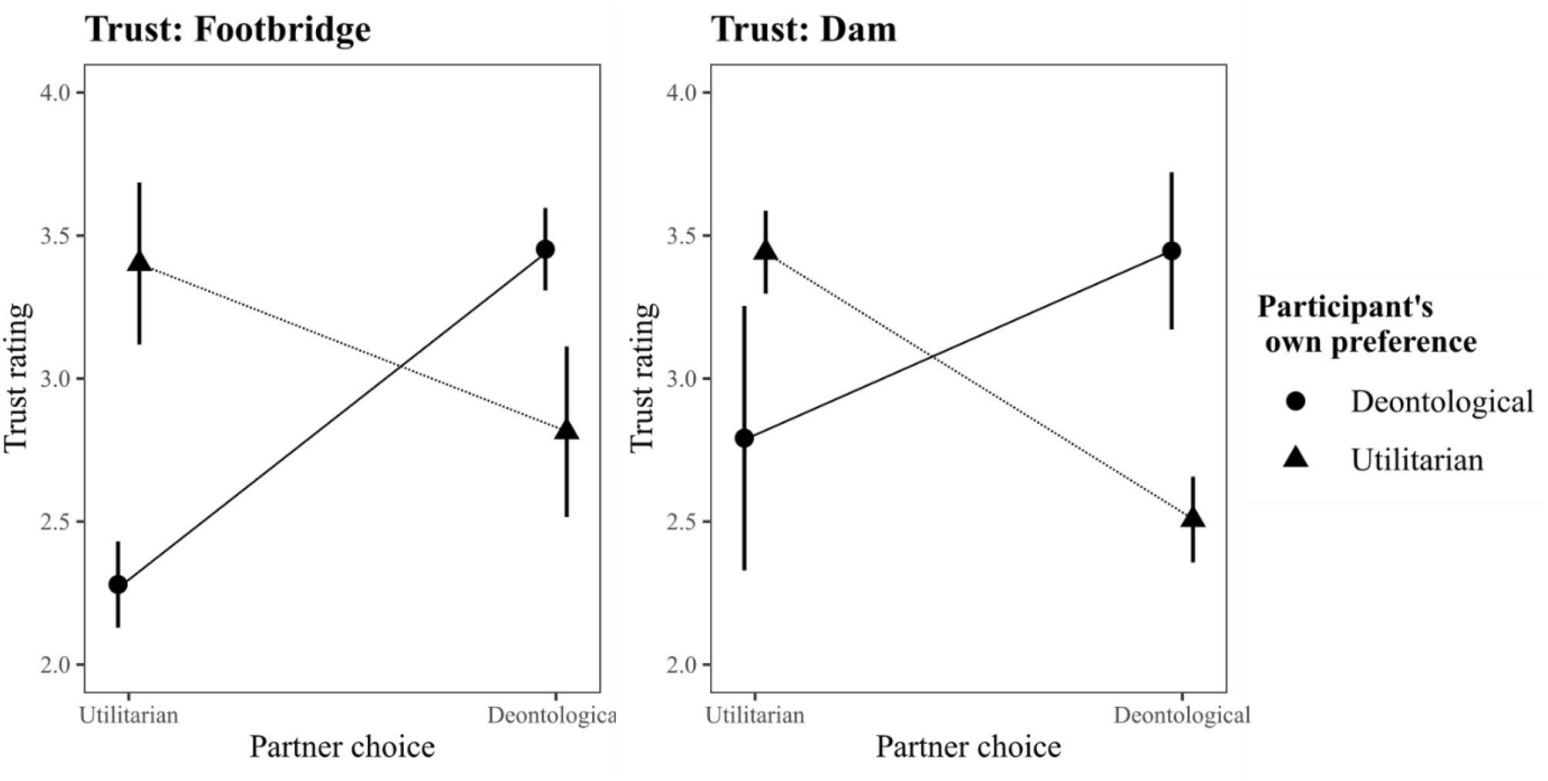
Inferred trust in the pilot study as a function of partner judgment and participants own moral preference for each of the two dilemmas included in that study.

Arguably, the methodology we have used is the biggest limitation of this work. A critical reader might note that we have demonstrated the effects we uncovered on only a single dilemma and point out that that dilemma had a majority utilitarian opinion. Whether our findings can be generalized to other dilemmas, and especially to dilemmas with a deontological majority opinion, is unclear. There is certainly merit to this concern. Future studies should expand on this work and explicitly test whether our results hold up in other dilemma contexts as well. That having said, the pilot study we ran included two other dilemmas: one dilemma with a utilitarian majority opinion (should you flood a small town to save a larger one), and one with a deontological majority opinion (should you push a stranger in front of a trolley-train to save five people, i.e., the footbridge dilemma). Re-analyzing the data from the pilot study demonstrates that the same pattern of results emerges in that study as well. As Fig. 4 demonstrates, participants’ own moral preference significantly interacts with Partner choice, and it does so on both dilemmas (both *p* < 0.001; full results available in the supplementary materials). Thus, even on some deontological majority dilemmas like the footbridge dilemma, utilitarians trust utilitarians, and deontologists trust deontologists.

The main result of the current work might strike readers as a little odd. Did previous work in this area not control for participants’ own preference? Some studies (e.g., Bostyn et al.^10^) controlled for participants’ moral preference by controlling for general attitudes towards utilitarian or deontological judgment, rather than controlling for the preference that participants display on the specific moral dilemma used in the trust perception task. That distinction might explain why the interaction uncovered in the current work did not arise in those studies. Other studies did control for preference on the specific moral dilemmas used in the trust perception task: for instance, the classic work by Everett et al.^7^ In that work, the interaction between personal preference and partner choice emerges in one of their studies but does not emerge in multiple others. Reasonably, Everett and colleagues concluded against the presence of the similarity effect we uncovered here. However, in later work by the same authors, the effect does appear. For instance, in all three studies from Everett et al.^39^ that included sacrificial moral dilemmas, the similarity effect also crops up. In those studies, just like ours, utilitarians trusted utilitarians and non-utilitarians trusted non-utilitarians. However, because the authors used sacrificial dilemmas on which the majority of people preferred the deontological option, they also found that people distrusted utilitarians on average, even when controlling for participants’ own judgments as a covariate. While future research will need to corroborate our findings, we think it possible that a focus on deontological majority dilemmas along with the early null findings have caused researchers in this area to dismiss the similarity hypothesis too prematurely.

## Conclusion

The current work suggests that it is imprudent to claim that deontologists are generally trusted over utilitarians. Our results indicate that whether that is the case could be context-dependent and contingent on the features of the dilemma in question or the personal moral preference of trustors. Arguably, this helps to resolve the paradox that policymakers face. Previous research has suggested that policymakers can risk losing public support if they pursue a course that minimizes harm through sacrificial harm^38^. Our results suggest that if policymakers follow the majority opinion, they will likely be sheltered from any potential negative consequences of their decision and might even gain support when a policy involving sacrificial harm is broadly supported. Rather than people displaying a general preference for one type of moral judgment, our results suggest that similarity might play a key role after all.

## Supplementary Information

Supplementary Information.

## Data Availability

All materials, and data are available on https://osf.io/xayu7.

## Code availability

All statistical code is available on https://osf.io/xayu7.
