# Supplementary material for: Deontologists are not always trusted over utilitarians: revisiting inferences of trustworthiness from moral judgments
Source: Sci Rep. 2023 Jan 30;13:1665. doi: 10.1038/s41598-023-27943-3 (PMC9885386; doi:10.1038/s41598-023-27943-3)
Supplement: Supplementary file 1 — Supplementary Information. [file 41598_2023_27943_MOESM1_ESM.docx]

# **Supplementary Information for:**

**Deontologists are not always trusted over utilitarians: Revisiting Inferences of Trustworthiness from Moral Judgments**

Dries H. Bostyn, Subramanya Prasad Chandrashekar, Arne Roets

*Correspondence to: [Dries.Bostyn@Ugent.be](mailto:Dries.Bostyn@Ugent.be)

# **Pilot Study**

**Ethical approval.**

The pilot study was approved by the Research Ethics Committee of the second author by the Research Ethics Committee of The Hong Kong Metropolitan University. For the pilot study, we invited participants older than 18 years. All the study procedures and methods noted in the MS are in accordance with the relevant guidelines and regulations. Informed consent was obtained from all participants of the Pilot study.

# **Pilot study: Method and Results**

***Participants***

We recruited a convenience sample of American participants (N = 300, after exclusions N = 272; females = 36, Mean age = 41.98) through the online labor platform CloudResearch in July 2021. Participants were excluded if they self-reported a low proficiency of English, self-reported not being serious about filling in the survey, failed the comprehension checks (see the section: baseline trust and cooperation), did not complete the survey. Sample size was not determined by an a priori power calculation but based on available funding and similar to those used in prior work on this topic (particularly, Bostyn et al., 2017; and Everett et al., 2016). A power sensitivity analysis demonstrates that the sample had 80% power to detect effects of size d ≥ 0.35 (assuming alpha = 0.05).

***Procedure***

Participants were invited to complete an online survey through the Qualtrics platform and were asked to provide their consent before taking part in the study. The experiment consisted of four tasks. First, participants were asked to complete two moral dilemmas that were presented in randomized order to gauge their own moral preferences (as a potential control measure). Secondly, they were asked to play an *uninformed* trust game with an unknown other (see infra) to measure their baseline levels of trust in unknown others. Thirdly, they played two *informed* trust games. Again, participants played these games with unknown others, but on each of these informed trust games they received some information about their partner in the trust game: the moral choice that partner had made on one of the two sacrificial dilemmas they had previously responded to. Across the two informed trust games, participants were presented again with both dilemmas presented in the first task. This allowed us to measure participants’ trust in those making deontological or utilitarian decisions. Finally, participants were asked how serious they were when completing the survey, whether they had seen the materials used in this study before, what they thought the purpose of the study was, to rate their understanding of the English used in the study, whether they had comments about how we could better run these types of studies and if they were satisfied with their payment as well as a series of questions aimed at measuring demographic variables (Age, Gender, Country of birth, subjective SES, Religion, Religiosity, ethnic background, political orientation).

***Materials and Measures***

**Moral preference task.** Participants were asked to complete two sacrificial dilemmas. The first dilemma described the footbridge dilemma in which people are asked whether it is morally appropriate to push a single man in front of a trolley-train to save five people from being runover. The second dilemma asked participants to imagine they were a governor dealing with an impending break in a water dam that would flood a large city with 10000 inhabitants. The scenario described that this crisis could only be averted by opening floodgates that would cause another smaller town (of 500 inhabitants) to be flooded instead. The text of both dilemmas are provided below.

**Footbridge dilemma:**

Imagine the following scenario. A runaway trolley is heading down the tracks toward five workers who will all be killed if the trolley proceeds on its present course. You are on a footbridge over the tracks, in between the approaching trolley and the five workers. Next to you on this footbridge is a stranger who happens to be very large. The only way to save the lives of the five workers is to push this stranger off the bridge and onto the tracks below where his large body will stop the trolley. The stranger will die if you does this, but the five workers will be saved.

**Dam dilemma:**

Imagine the following scenario. You are the governor of a state that was just hit by a hurricane. The hurricane has damaged a dam causing it to be on the verge of breaking down. When the dam breaks, the water will flood a nearby town with 10,000 inhabitants. While the break cannot be averted, you could open a floodgate to divert the water to another river thus saving the city from a flood. However, doing so will flood another, smaller town with 500 inhabitants. Fortunately, you have already managed to evacuate both towns to ensure that no lives will be lost. Do you decide not to do anything, which would cause a town of 10,000 people to be flooded, or do you divert the water, which would cause a flooding in a town of 500 people.

Participants’ moral preferences were measured in two ways: first, participants were asked whether they would opt for the sacrificial harm in a binary manner (Yes or No). Subsequently, they were asked to rate how morally appropriate the sacrificial harm was on a seven-point scale going from (1) “*Absolutely Inappropriate*” to (7) “*Absolutely Appropriate*”.

**Baseline trust and cooperation.** To measure baseline levels of trust and cooperation, participants were asked to play a hypothetical trust game with an unknown other (for a similar procedure see Bostyn & Roets, 2017). This trust game was structured as follows: all participants were informed they would be playing the game from the perspective of a ‘trustor’. Trustors were informed they would receive 10 credits and could freely decide how many of these they wanted to give to a 'trustee'. They were also informed that all credits given to the 'trustee' would be tripled and that the 'trustee' would then subsequently decide how much of this sum they wanted to give back to the 'trustor'. All participants were informed that they would be playing three trust games in total, the first of which was aimed at measuring their baseline trust. Participants were told that they would not be playing these trust games "live” but that they were playing with people that had previously completed the survey and that their responses would be linked together after data collection was completed. The detailed descriptions of the trust game are reported in the ‘**Pilot study materials section**.’

After receiving instructions on the trust game, participants were asked two comprehension questions. First, they were asked to imagine that a trustor transfers 5 credits to the trustee and that the trustee then has to decide how many credits they want to return. Participants were asked how much the trustee should return if the trustee wanted to maximize their own credits, and were given four choice options: 0, 5, 10, or 15. Subsequently, they were asked how many credits a trustee would receive if a trustor endows them with 10 credits and presented with another four choice options: 10, 20, 30, and 40 credits.

After completing the comprehension checks, participants were told they were matched up with a random trustee. Participants were asked to rate how much they trusted this trustee on a five-point scale going from (1) “*Not at all*” to (5) “*Completely*” and were asked how many credits they wanted to give to this trustee through a sliding scale spanning from 0 to 10.

Before completing the trust games, participants were also informed that their choices on these games would impact a bonus they could earn spanning from US$ 0.05 to US$ 0.25. We did not specify how these bonuses would be determined, assuming that participants would think that bonuses would be determined by the outcome of the trust games. In reality, their bonuses were determined as a linear function of how much they trusted the unknown other participant in the trust game and scores on the 5-point trust scale were multiplied by 5 cents to determine participant bonuses.

**Trust in and cooperation with utilitarian and deontological others.** After completing our measure for baseline trust, participants played two more trust games to measure trust in utilitarian and deontological others. Again, participants played these trust games from the perspective of the trustor. Before each of these games, participants were informed that they were playing this game with a different partner. On each of these games, participants were presented with one of the two moral dilemmas they had previously been confronted with (the footbridge and dam dilemmas) and told their partner in the trust game had decided either in favor of the deontological or utilitarian option. Whether participants were confronted with deontological or utilitarian partners was randomly determined and manipulated between-subjects. Participants either played both trust games with a deontological partner or both trust games with a utilitarian partner. Participants were asked to what extent they trusted this partner, as well as how many credits they would give to this partner using the same scales as above.

***Results***

Before analyzing participants’ responses to the trust games, we investigated how they themselves responded to the two dilemmas we presented them with. As expected based on prior studies, 81% of the study participants favored the deontological decision when answering the footbridge dilemma (Greene et al., 2001). In contrast, when confronted with the dam dilemma, only 14% of participants decided in favor of the deontological option (See Figure S1). A two-sided proportion test confirmed that the proportion of deontological choices on the footbridge dilemma was higher than in the dam dilemma, 𝜒^2^(1) = 252.16, *p < .*001.

***Figure S1*.** Proportions of deontological and utilitarian responses for each dilemma.


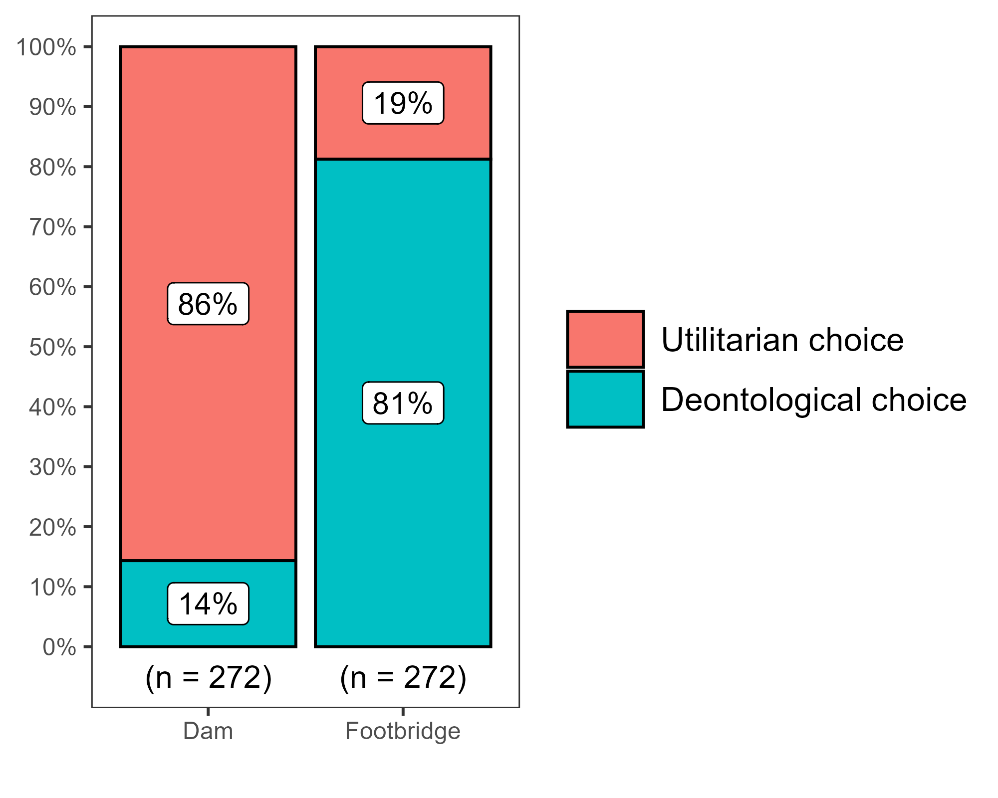


**Table S1.** **Results of Pilot study:** Descriptive tables.

| **Variables** | *n* | Mean | S.D. |
| --- | --- | --- | --- |
| **Trust ratings** | | | |
| Baseline trust | 272 | 2.41 | 0.95 |
| Trust toward Deontological partner | 134 | 2.72 | 1.00 |
| Trust toward utilitarian partner | 138 | 2.54 | 1.12 |
| **Credit transferred** | | | |
| Baseline credit transfer | 272 | 5.73 | 3.59 |
| Credit transfer toward Deontological partner | 134 | 5.81 | 3.52 |
| Credit transfer toward utilitarian partner | 138 | 5.15 | 3.84 |

*Note.* The descriptive statistics of ratings grouped by outcome (deontological partner, utilitarian partner) include responses to both Footbridge and Dam dilemmas.

Subsequently, we turned to the question of whether participants trusted deontologists over utilitarians. We conducted two sets of linear mixed-effects models: one with trust ratings as the outcome measure, and the second with the amount of credits transferred. On both models, we included participants’ own moral preference, their base trust ratings or credit transfers (depending on the outcome measure) as control measures. More importantly, we also included the type of moral decision by their partner (Utilitarian vs. Deontological), vignette type (Footbridge vs. Dam), as well as the interaction between these latter two variables as fixed effect predictors. On both models we included participant ID as a random intercept.

Results were consistent across both sets of regression analysis. Crucially, we found that interaction terms were significant, both |*t*| > 9.79, *p* <.001 (See Table S2 & Table S3). Corroborating earlier studies, these models demonstrated that deontological partners were trusted more and received higher amounts of credits than utilitarian partners in the footbridge vignette condition (See Figure S2). However, on the dam vignette we found the opposite: utilitarian partners were trusted more and received higher amounts of credits than deontological partners. These results are summarized in Figure and hold independently of whether we control for participants own moral preferences or not.

Table S2. Results of Pilot study: Estimated fixed-effects coefficients of the mixed-effects regression model with trust ratings as the DV.

| **Predictors** | **DV: Trust ratings** | | | | |
| --- | --- | --- | --- | --- | --- |
|  | **Estimates** | **std. Error** | **CI** | **t-statistic** | ***p*** |
| Intercept | 0.89 | 0.15 | 0.60 – 1.17 | 6.08 | **<.001** |
| Participant's moral preference | -0.02 | 0.09 | -0.20 – 0.16 | -0.26 | .797 |
| Baseline trust | 0.61 | 0.04 | 0.53 – 0.69 | 14.58 | **<.001** |
| Vignette (Footbridge vs. Dam) | 0.64 | 0.1 | 0.45 – 0.84 | 6.48 | **<.001** |
| Outcome condition (Utilitarian vs Deontological) | 0.68 | 0.1 | 0.49 – 0.88 | 6.88 | **<.001** |
| Vignette x Outcome condition | -1.51 | 0.12 | -1.74 – -1.29 | -13.02 | **<.001** |

Table S3. Results of Pilot study: Estimated fixed-effects coefficients of the mixed-effects regression model with the amount of credit transferred as the DV.

| **Predictors** | **DV: Credit transfer** | | | | |
| --- | --- | --- | --- | --- | --- |
|  | **Estimates** | **S.E.** | **CI** | **t-statistic** | ***p*** |
| Intercept | 0.23 | 0.31 | -0.38 – 0.84 | 0.73 | **.464** |
| Participant's moral preference | -0.23 | 0.24 | -0.71 – 0.24 | -0.96 | **.337** |
| Baseline credit transfer | 0.82 | 0.03 | 0.76 – 0.87 | 29.4 | **<.001** |
| Vignette (Footbridge vs. Dam) | 1.41 | 0.27 | 0.88 – 1.95 | 5.16 | **<.001** |
| Outcome condition (Utilitarian vs Deontological) | 1.54 | 0.26 | 1.03 – 2.04 | 5.93 | **<.001** |
| Vignette x Outcome condition | -3.18 | 0.33 | -3.82 – -2.55 | -9.79 | **<.001** |

***Figure S2*.** Inferred trust and cooperation in Pilot study as a function of partner judgment and vignette type.


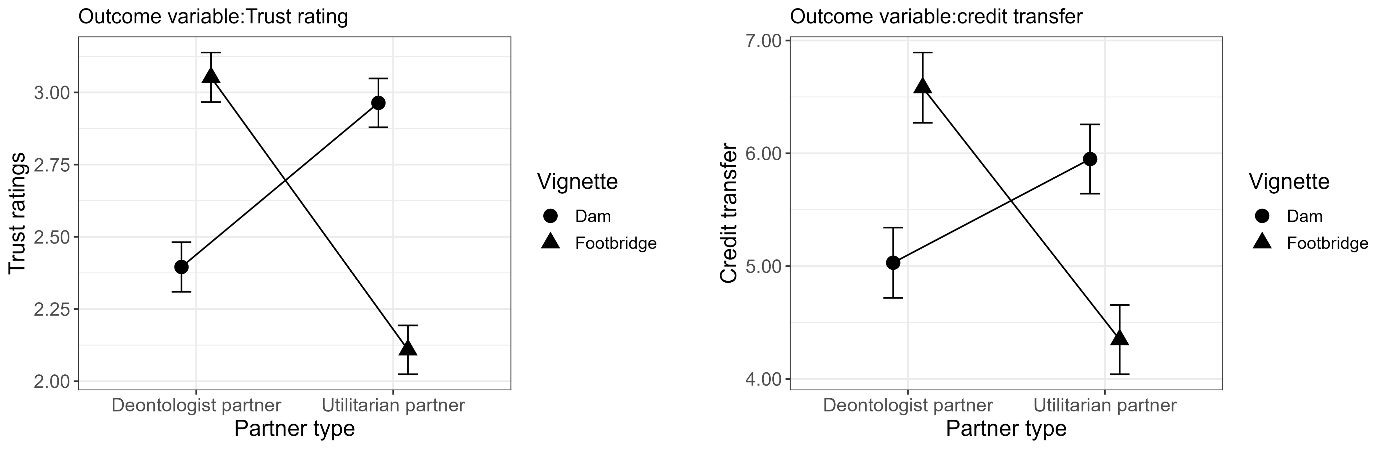


# **Supplementary Analyses: Main Study**

# **Replication analyses**

Table S4*. Effects of Partner Choice and participants’ Own preference. Categorical variables were dummy coded. Top line displays the result using the sample with preregistered exclusions. Bottom line displays results using the full sample.*

| Trust ~ | $\hat{b}$ | *se* | *df* | *t* | *p* |
| --- | --- | --- | --- | --- | --- |
| Intercept | 1.84 | 0.03 | 1300 | 70.21 | <.001 |
|  | 1.99 | 0.02 | 1958 | 87.28 | <.001 |
| Partner Choice: Utilitarian | 1.22 | 0.02 | 6532 | 68.74 | <.001 |
|  | 1.13 | 0.02 | 9829 | 73.56 | <.001 |
| Own: Deontological | 1.08 | 0.03 | 7256 | 32.32 | <.001 |
|  | 0.99 | 0.03 | 10811 | 36.33 | <.001 |
| Partner Choice x Own | -2.01 | 0.04 | 6620 | -48.89 | <.001 |
|  | -1.76 | 0.03 | 9965 | -51.82 | <.001 |

| Credits ~ | $\hat{b}$ | *se* | *df* | *t* | *p* |
| --- | --- | --- | --- | --- | --- |
| Intercept | 3.36 | 0.11 | 1071 | 30.57 | <.001 |
|  | 3.55 | 0.08 | 1643 | 41.83 | <.001 |
| Partner Choice: Utilitarian | 2.66 | 0.05 | 6517 | 55.12 | <.001 |
|  | 2.46 | 0.04 | 9809 | 62.38 | <.001 |
| Own preference: Deontological | 2.22 | 0.09 | 6872 | 23.88 | <.001 |
|  | 2.00 | 0.07 | 10324 | 28.23 | <.001 |
| Partner Choice x Own | -4.17 | 0.11 | 6651 | -37.12 | <.001 |
|  | -3.70 | 0.09 | 9869 | -42.25 | .<001 |

*Note.* Coding of the dummy variables: 1) Partner choice: 1= Utilitarian, 0 = Deontological; 1) Own preference (i.e., participant's own preference): 0= Utilitarian, 1 = Deontological.

Table S5*. Effects of Partner Choice and participants’ Own preference controlled for Base levels of trust and cooperation. Categorical variables were dummy coded. Top line displays the result using the sample with preregistered exclusions. Bottom line displays results using the full sample.*

| Trust ~ | $\hat{b}$ | *se* | *df* | *t* | *p* |
| --- | --- | --- | --- | --- | --- |
| Intercept | 0.71 | 0.05 | 1020 | 15.20 | <.001 |
|  | 0.72 | 0.04 | 1523 | 18.38 | <.001 |
| Partner Choice: Utilitarian | 1.22 | 0.02 | 6549 | 68.77 | <.001 |
|  | 1.13 | 0.02 | 9868 | 73.62 | <.001 |
| Own: Deontological | 1.09 | 0.03 | 7431 | 33.57 | <.001 |
|  | 0.98 | 0.03 | 11160 | 37.20 | <.001 |
| Base Trust | 0.48 | 0.02 | 926 | 27.10 | <.001 |
|  | 0.51 | 0.01 | 1397 | 35.79 | <.001 |
| Partner Choice x Own | -2.01 | 0.04 | 6703 | -48.98 | <.001 |
|  | -1.76 | 0.03 | 10122 | -52.01 | <.001 |

| Credits ~ | $\hat{b}$ | *se* | *df* | *T* | *p* |
| --- | --- | --- | --- | --- | --- |
| Intercept | -0.62 | 0.10 | 1083 | -6.12 | <.001 |
|  | -0.43 | 0.09 | 1609 | -4.99 | <.001 |
| Partner Choice: Utilitarian | 2.66 | 0.05 | 6540 | 55.14 | <.001 |
|  | 2.45 | 0.04 | 9850 | 29.64 | <.001 |
| Own preference: Deontological | 2.27 | 0.09 | 7381 | 25.46 | <.001 |
|  | 2.04 | 0.07 | 11038 | 29.64 | <.001 |
| Base Credit | 0.74 | 0.01 | 925 | 49.70 | <.001 |
|  | 0.73 | 0.01 | 1394 | 56.94 | <.001 |
| Partner Choice x Own | -4.15 | 0.11 | 6660 | -37.13 | <.001 |
|  | -3.68 | 0.09 | 10043 | -42.26 | .<001 |

*Note.* Coding of the dummy variables: 1) Partner choice: 1= Utilitarian, 0 = Deontological; 1) Own preference (i.e., participant's own preference): 0= Utilitarian, 1 = Deontological.

**Main analyses**

Table S6*. Effects of the Action-feature.* *Categorical variables were dummy coded. Top line displays the result using the sample with preregistered exclusions. Bottom line displays results using the full sample.*

| Trust ~ | $\hat{b}$ | *se* | *df* | *t* | *p* |
| --- | --- | --- | --- | --- | --- |
| Intercept | 0.73 | 0.05 | 1213 | 15.09 | <.001 |
|  | 0.78 | 0.04 | 1826 | 18.80 | <.001 |
| Partner Choice: Utilitarian | 1.22 | 0.03 | 6693 | 45.87 | <.001 |
|  | 1.10 | 0.03 | 10087 | 48.34 | <.001 |
| Own: Deontological | 1.07 | 0.05 | 7373 | 26.27 | <.001 |
|  | 0.97 | 0.04 | 11021 | 28.68 | <.001 |
| Inaction | -0.05 | 0.03 | 6874 | -1.87 | .061 |
|  | -0.10 | 0.02 | 10340 | -4.17 | <.001 |
| Base trust | 0.48 | 0.02 | 926 | 27.12 | <.001 |
|  | 0.51 | 0.01 | 1398 | 35.80 | <.001 |
| Partner Choice: Uti * Own: Deo | -2.04 | 0.05 | 6741 | -38.52 | <.001 |
|  | -1.84 | 0.05 | 10170 | -40.60 | <.001 |
| Partner Choice: Uti * Inaction | -0.01 | 0.04 | 6824 | -0.26 | .798 |
|  | 0.05 | 0.03 | 10283 | 1.65 | .100 |
| Own: Deo * Inaction | 0.02 | 0.06 | 6999 | 0.29 | .774 |
|  | -0.02 | 0.05 | 10533 | -0.33 | .743 |
| Partner Choice * Own * Inaction | 0.07 | 0.09 | 6803 | 0.86 | .393 |
|  | 0.20 | 0.07 | 10231 | 2.87 | .004 |

| Credits ~ | $\hat{b}$ | *se* | *df* | *t* | *p* |
| --- | --- | --- | --- | --- | --- |
| Intercept | -0.57 | 0.11 | 1419 | -5.29 | <.001 |
|  | -0.33 | 0.09 | 2045 | -3.55 | <.001 |
| Partner Choice: Utilitarian | 2.65 | 0.07 | 6652 | 36.49 | <.001 |
|  | 2.37 | 0.06 | 10016 | 40.50 | <.001 |
| Own preference: Deontological | 2.24 | 0.11 | 7286 | 20.03 | <.001 |
|  | 2.02 | 0.09 | 10849 | 22.87 | <.001 |
| Inaction | -0.08 | 0.07 | 6801 | -1.05 | .293 |
|  | -0.20 | 0.06 | 10216 | -3.29 | .<001 |
| Base trust | 0.74 | 0.01 | 925 | 49.70 | <.001 |
|  | 0.73 | 0.01 | 1394 | 56.96 | <.001 |
| Partner Choice × Own preference | -4.12 | 0.14 | 6690 | -28.68 | <.001 |
|  | -3.74 | 0.12 | 10080 | -32.13 | <.001 |
| Partner Choice × Inaction | 0.01 | 0.10 | 6758 | 0.10 | .918 |
|  | 0.16 | 0.08 | 10169 | 1.89 | .059 |
| Own: Deo × Inaction | 0.03 | 0.17 | 6897 | 0.21 | .836 |
|  | -0.03 | 0.13 | 10378 | -0.22 | .826 |
| Partner Choice × Own × Inaction | -0.04 | 0.24 | 6740 | -0.18 | .854 |
|  | 0.20 | 0.18 | 10127 | 1.10 | .273 |

*Note.* Coding of the dummy variables: 1) Partner choice: 1= Utilitarian, 0 = Deontological; 1) Own preference (i.e., participant's own preference): 0= Utilitarian, 1 = Deontological; Action frame: 1= Inaction, 0= Action.

Table S7. *Effects of the Instrumentality-feature.* *Categorical variables were dummy coded. Top line displays the result using the sample with preregistered exclusions. Bottom line displays results using the full sample.*

| Trust ~ | $\hat{b}$ | *se* | *df* | *t* | *p* |
| --- | --- | --- | --- | --- | --- |
| Intercept | 0.71 | 0.05 | 1179 | 14.66 | <.001 |
|  | 0.74 | 0.04 | 1777 | 17.93 | <.001 |
| Partner Choice: Utilitarian | 1.22 | 0.03 | 6713 | 47.24 | <.001 |
|  | 1.12 | 0.02 | 10114 | 50.47 | <.001 |
| Own: Deontological | 1.09 | 0.04 | 7276 | 25.25 | <.001 |
|  | 0.99 | 0.04 | 10889 | 27.88 | <.001 |
| Instrumental: Yes | -0.00 | 0.03 | 6815 | -0.05 | .959 |
|  | -0.02 | 0.02 | 10260 | -0.94 | .348 |
| Base trust | 0.48 | 0.02 | 926 | 27.08 | <.001 |
|  | 0.51 | 0.01 | 1397 | 35.78 | <.001 |
| Partner Choice × Own | -2.05 | 0.06 | 6743 | -35.23 | <.001 |
|  | -1.82 | 0.05 | 10196 | -37.70 | <.001 |
| Partner Choice × Instrumental | -0.01 | 0.04 | 6854 | -0.16 | .872 |
|  | 0.01 | 0.03 | 10326 | 0.38 | .701 |
| Own × Instrumental | -0.00 | 0.06 | 6811 | -0.04 | .970 |
|  | -0.02 | 0.04 | 10276 | -0.33 | .743 |
| Partner Choice × Own × Instrumental | 0.08 | 0.08 | 6823 | 1.01 | .315 |
|  | 0.11 | 0.07 | 10311 | 1.56 | .119 |

| Credits ~ | $\hat{b}$ | *se* | *df* | *t* | *p* |
| --- | --- | --- | --- | --- | --- |
| Intercept | -0.63 | 0.11 | 1367 | -5.90 | <.001 |
|  | -0.42 | 0.09 | 1995 | -4.65 | <.001 |
| Partner Choice: Utilitarian | 2.65 | 0.07 | 6667 | 37.67 | <.001 |
|  | 2.42 | 0.06 | 10036 | 42.36 | <.001 |
| Own: Deontological | 2.30 | 0.12 | 7178 | 19.46 | <.001 |
|  | 2.05 | 0.09 | 10719 | 22.31 | <.001 |
| Instrumental: Yes | 0.03 | 0.07 | 6749 | 0.45 | .651 |
|  | -0.01 | 0.06 | 10149 | -0.17 | .863 |
| Base trust | 0.74 | 0.01 | 926 | 49.68 | <.001 |
|  | 0.73 | 0.01 | 1395 | 56.91 | <.001 |
| Partner Choice × Own | -4.19 | 0.16 | 6693 | -26.42 | <.001 |
|  | -3.72 | 0.12 | 10100 | -30.06 | <.001 |
| Partner Choice × Instrumental | 0.00 | 0.10 | 6780 | 0.04 | .968 |
|  | 0.06 | 0.08 | 10199 | 0.770 | .442 |
| Own × Instrumental | -0.06 | 0.16 | 6747 | -0.37 | .712 |
|  | -0.03 | 0.12 | 10162 | -0.26 | .795 |
| Partner Choice × Own × Instrumental | 0.09 | 0.23 | 6758 | 0.38 | .702 |
|  | 0.09 | 0.18 | 10190 | 0.50 | .617 |

*Note.* Coding of the dummy variables: 1) Partner choice: 1= Utilitarian, 0 = Deontological; 1) Own preference (i.e., participant's own preference): 0= Utilitarian, 1 = Deontological; Instrumental: 1= Yes, 0= No.

Table S8. *Effects of Cause-feature.* *Categorical variables were dummy coded. Top line displays the result using the sample with preregistered exclusions. Bottom line displays results using the full sample.*

| Trust ~ | $\hat{b}$ | *se* | *df* | *t* | *p* |
| --- | --- | --- | --- | --- | --- |
| Intercept | 0.70 | 0.05 | 1182 | 14.46 | <.001 |
|  | 0.72 | 0.04 | 1780 | 17.56 | <.001 |
| Partner Choice: Uti | 1.26 | 0.03 | 6715 | 48.59 | <.001 |
|  | 1.14 | 0.02 | 10117 | 51.13 | <.001 |
| Own: Deo | 1.06 | 0.04 | 7293 | 24.25 | <.001 |
|  | 0.96 | 0.04 | 10921 | 27.11 | <.001 |
| Caused: Yes | 0.02 | 0.03 | 6814 | 0.66 | .513 |
|  | 0.01 | 0.02 | 10258 | 0.34 | .736 |
| Base trust | 0.48 | 0.02 | 926 | 27.10 | <.001 |
|  | 0.51 | 0.01 | 1397 | 35.78 | <.001 |
| Partner Choice: Uti * Own: Deo | -2.03 | 0.06 | 6760 | -34.67 | <.001 |
|  | -1.78 | 0.05 | 10211 | -36.67 | <.001 |
| Partner Choice: Uti * Caused | -0.07 | 0.04 | 6864 | -2.00 | .045 |
|  | -0.03 | 0.03 | 10337 | -0.86 | .388 |
| Own: Deo * Caused | 0.06 | 0.06 | 6827 | 1.08 | .282 |
|  | 0.05 | 0.05 | 10277 | 0.99 | .321 |
| Partner Choice * Own * Caused | 0.04 | 0.08 | 6864 | 0.49 | .626 |
|  | 0.04 | 0.07 | 10349 | 0.58 | .563 |

| Credits ~ | $\hat{b}$ | *se* | *df* | *t* | *p* |
| --- | --- | --- | --- | --- | --- |
| Intercept | -0.23 | 0.24 | 1000 | -0.97 | .331 |
|  | 0.22 | 0.19 | 1510 | 1.18 | .239 |
| Partner Choice: Uti | 2.67 | 0.07 | 6565 | 37.78 | <.001 |
|  | 2.43 | 0.06 | 9889 | 42.15 | <.001 |
| Own: Deo | 2.08 | 0.12 | 6823 | 17.02 | <.001 |
|  | 1.83 | 0.09 | 10247 | 19.62 | <.001 |
| Caused | -0.05 | 0.07 | 6595 | -0.674 | .501 |
|  | -0.09 | 0.06 | 9933 | -1.59 | .112 |
| Base trust | 1.53 | 0.09 | 928 | 16.74 | <.001 |
|  | 1.35 | 0.07 | 1399 | 20.05 | <.001 |
| Partner Choice: Uti * Own: Deo | -4.15 | 0.16 | 6578 | -25.86 | <.001 |
|  | -3.63 | 0.13 | 9918 | -28.88 | <.001 |
| Partner Choice: Uti * Caused | -0.03 | 0.10 | 6611 | -0.28 | .783 |
|  | 0.05 | 0.08 | 9960 | 0.60 | .548 |
| Own: Deo * Caused | 0.28 | 0.16 | 6599 | 1.77 | .077 |
|  | 0.30 | 0.12 | 9939 | 2.46 | .014 |
| Partner Choice * Own * Caused | -0.03 | 0.23 | 6611 | -0.13 | .900 |
|  | -0.14 | 0.18 | 9964 | -0.75 | .451 |

*Note.* Coding of the dummy variables: 1) Partner choice: 1= Utilitarian, 0 = Deontological; 1) Own preference (i.e., participant's own preference): 0= Utilitarian, 1 = Deontological; Causality: 1= Yes, 0= No.

Table S9. *Effects of the Mortality-feature.* *Categorical variables were dummy coded. Top line displays the result using the sample with preregistered exclusions. Bottom line displays results using the full sample.*

| Trust ~ | $\hat{b}$ | *se* | *df* | *t* | *p* |
| --- | --- | --- | --- | --- | --- |
| Intercept | 0.76 | 0.05 | 1214 | 15.60 | <.001 |
|  | 0.77 | 0.04 | 1810 | 18.71 | <.001 |
| Partner Choice: Uti | 1.16 | 0.03 | 6670 | 44.43 | <.001 |
|  | 1.08 | 0.02 | 10099 | 48.09 | <.001 |
| Own: Deo | 1.06 | 0.04 | 7275 | 24.56 | <.001 |
|  | 0.95 | 0.03 | 10930 | -3.87 | <.001 |
| Mortality: Yes | -0.10 | 0.03 | 6799 | -3.62 | <.001 |
|  | -0.09 | 0.02 | 10261 | -3.87 | <.001 |
| Base trust | 0.48 | 0.02 | 926 | 27.11 | <.001 |
|  | 0.51 | 0.01 | 1397 | 35.81 | <.001 |
| Partner Choice: Uti * Own: Deo | -1.96 | 0.06 | 6754 | -33.81 | <.001 |
|  | -1.71 | 0.05 | 10180 | -36.48 | <.001 |
| Partner Choice: Uti * Mortality | 0.10 | 0.04 | 6830 | 2.73 | .006 |
|  | 0.09 | 0.03 | 10298 | 2.65 | <.001 |
| Own: Deo * Mortality | 0.04 | 0.06 | 6875 | 0.76 | .450 |
|  | 0.05 | 0.04 | 10361 | 1.12 | .262 |
| Partner Choice * Own * Mortal. | -0.09 | 0.08 | 6817 | -1.13 | .260 |
|  | -0.10 | 0.07 | 10282 | -1.45 | .147 |

| Credits ~ | $\hat{b}$ | *se* | *df* | *t* | *P* |
| --- | --- | --- | --- | --- | --- |
| Intercept | -0.49 | 0.11 | 1415 | -4.52 | <.001 |
|  | -0.32 | 0.09 | 2033 | -3.45 | <.001 |
| Partner Choice: Uti | 2.54 | 0.07 | 6658 | 35.63 | <.001 |
|  | 2.36 | 0.06 | 10025 | 40.69 | <.001 |
| Own: Deo | 2.20 | 0.12 | 7176 | 18.56 | <.001 |
|  | 1.96 | 0.09 | 10758 | 27.73 | <.001 |
| Mortality | -0.24 | 0.07 | 6737 | -3.27 | .001 |
|  | -0.22 | 0.06 | 10152 | -3.66 | <.001 |
| Base trust | 0.74 | 0.01 | 925 | 49.70 | <.001 |
|  | 0.73 | 0.01 | 1394 | 56.85 | <.001 |
| Partner Choice: Uti * Own: Deo | -4.11 | 0.16 | 6701 | -26.00 | <.001 |
|  | -3.60 | 0.12 | 10087 | -29.87 | <.001 |
| Partner Choice: Uti * Mortality | 0.21 | 0.10 | 6762 | 2.07 | .038 |
|  | 0.18 | 0.08 | 10180 | 2.24 | .025 |
| Own: Deo * Mortality | 0.12 | 0.16 | 6799 | 0.78 | .437 |
|  | 0.13 | 0.12 | 10230 | 1.07 | .284 |
| Partner Choice * Own * Mortal. | -0.06 | 0.23 | 6751 | -0.28 | .777 |
|  | -0.14 | 0.18 | 10168 | -0.79 | .430 |

*Note.* Coding of the dummy variables: 1) Partner choice: 1= Utilitarian, 0 = Deontological; 1) Own preference (i.e., participant's own preference): 0= Utilitarian, 1 = Deontological; Mortal harm: 1= Yes, 0= No.

Table S10. *Effects of the Size-feature.* *Categorical variables were dummy coded. Top line displays the result using the sample with preregistered exclusions. Bottom line displays results using the full sample.*

| Trust ~ | $\hat{b}$ | *se* | *df* | *t* | *p* |
| --- | --- | --- | --- | --- | --- |
| Intercept | 0.65 | 0.05 | 1166 | 13.39 | <.001 |
|  | 0.66 | 0.04 | 10087 | 54.42 | <.001 |
| Partner Choice: Uti | 1.31 | 0.03 | 6695 | 51.44 | <.001 |
|  | 1.20 | 0.02 | 10087 | 54.52 | <.001 |
| Own: Deo | 1.19 | 0.05 | 7277 | 25.79 | <.001 |
|  | 1.08 | 0.04 | 10903 | 28.79 | <.001 |
| Size: Small | 0.13 | 0.03 | 6775 | 5.07 | <.001 |
|  | 0.13 | 0.02 | 10219 | 5.84 | <.001 |
| Base trust | 0.48 | 0.02 | 926 | 27.07 | <.001 |
|  | 0.51 | 0.01 | 1397 | 35.80 | <.001 |
| Partner Choice: Uti * Own: Deo | -2.09 | 0.06 | 6759 | -33.65 | <.001 |
|  | -1.81 | 0.05 | 10231 | -35.69 | <.001 |
| Partner Choice: Uti * Small | -0.18 | 0.04 | 6825 | -4.87 | <.001 |
|  | -0.14 | 0.03 | 10282 | -4.33 | <.001 |
| Own: Deo * Small | -0.19 | 0.06 | 6824 | -3.36 | <.001 |
|  | -0.19 | 0.05 | 10295 | -3.95 | <.001 |
| Partner Choice * Own * Small | 0.16 | 0.08 | 6814 | 1.93 | .054 |
|  | 0.10 | 0.07 | 10303 | 1.44 | .151 |

| Credits ~ | $\hat{b}$ | *se* | *df* | *t* | *P* |
| --- | --- | --- | --- | --- | --- |
| Intercept | -0.79 | 0.11 | 1333 | -7.38 | <.001 |
|  | -0.60 | 0.09 | 1959 | -6.59 | <.001 |
| Partner Choice: Uti | 2.93 | 0.07 | 6653 | 42.32 | <.001 |
|  | 2.68 | 0.06 | 10014 | 47.50 | <.001 |
| Own: Deo | 2.52 | 0.13 | 7175 | 19.94 | <.001 |
|  | 2.30 | 0.10 | 10729 | 23.68 | <.001 |
| Size: Small | 0.36 | 0.07 | 6717 | 4.99 | <.001 |
|  | 0.35 | 0.06 | 10115 | 5.92 | <.001 |
| Base trust | 0.74 | 0.01 | 925 | 49.54 | <.001 |
|  | 0.73 | 0.01 | 1394 | 56.82 | <.001 |
| Partner Choice: Uti * Own: Deo | -4.38 | 0.17 | 6704 | -25.92 | <.001 |
|  | -3.92 | 0.13 | 10125 | -30.04 | <.001 |
| Partner Choice: Uti * Small | -0.55 | 0.10 | 6757 | -5.45 | <.001 |
|  | -0.46 | 0.08 | 10164 | -5.64 | <.001 |
| Own: Deo * Small | -0.48 | 0.16 | 6756 | -3.05 | .002 |
|  | -0.51 | 0.12 | 10176 | -4.11 | <.001 |
| Partner Choice * Own * Small | 0.48 | 0.23 | 6748 | 2.10 | .036 |
|  | 0.47 | 0.18 | 10182 | 2.65 | .008 |

*Note.* Coding of the dummy variables: 1) Partner choice: 1= Utilitarian, 0 = Deontological; 1) Own preference (i.e., participant's own preference): 0= Utilitarian, 1 = Deontological; Size: 1= Small, 0= Large.

**Supplementary Analyses: Pilot Study**

Table S11*. Effects of Partner Choice and participants’ Own preference on the Footbridge dilemma used in the Pilot. Categorical variables were dummy coded.*

| Trust ~ | $\hat{b}$ | *se* | *df* | *t* | *p* |
| --- | --- | --- | --- | --- | --- |
| Intercept | 1.71 | 0.14 | 267 | 12.43 | <.001 |
| Partner Choice: Utilitarian | -1.17 | 0.10 | 267 | -11.80 | <.001 |
| Own: Utilitarian | -0.64 | 0.17 | 267 | -3.85 | <.001 |
| Base Trust | 0.58 | 0.05 | 267 | 12.33 | <.001 |
| Partner Choice x Own | 1.76 | 0.23 | 267 | 7.70 | <.001 |

| Credits ~ | $\hat{b}$ | *se* | *df* | *t* | *p* |
| --- | --- | --- | --- | --- | --- |
| Intercept | 2.13 | 0.30 | 267 | 7.22 | <.001 |
| Partner Choice: Utilitarian | -2.39 | 0.28 | 267 | -8.51 | <.001 |
| Own preference: Deontological | -1.73 | 0.47 | 267 | -3.71 | <.001 |
| Base Credit | 0.78 | 0.04 | 267 | 22.15 | <.001 |
| Partner Choice x Own | 3.77 | 0.65 | 267 | 5.85 | <.001 |

*Note.* Coding of the dummy variables: 1) Partner choice: 1 = Utilitarian, 0 = Deontological; 1) Own preference (i.e., participant's own preference): 1 = Utilitarian, 0 = Deontological.

Table S12*. Effects of Partner Choice and participants’ Own preference on the Dam dilemma used in the Pilot. Categorical variables were dummy coded.*

| Trust ~ | $\hat{b}$ | *se* | *df* | *t* | *p* |
| --- | --- | --- | --- | --- | --- |
| Intercept | 1.53 | 0.18 | 267 | 8.34 | <.001 |
| Partner Choice: Utilitarian | -0.65 | 0.27 | 267 | -2.40 | .017 |
| Own: Utilitarian | -0.94 | 0.16 | 267 | -6.02 | <.001 |
| Base Trust | 0.64 | 0.05 | 267 | 13.37 | <.001 |
| Partner Choice x Own | 1.59 | 0.29 | 267 | 5.47 | <.001 |

| Credits ~ | $\hat{b}$ | *se* | *df* | *t* | *p* |
| --- | --- | --- | --- | --- | --- |
| Intercept | 1.41 | 0.40 | 267 | 3.51 | <.001 |
| Partner Choice: Utilitarian | -1.36 | 0.71 | 267 | -1.93 | .055 |
| Own preference: Deontological | -1.99 | 0.41 | 267 | -4.91 | <.001 |
| Base Credit | 0.85 | 0.03 | 267 | 25.83 | <.001 |
| Partner Choice x Own | 3.42 | 0.75 | 267 | 4.55 | <.001 |

*Note.* Coding of the dummy variables: 1) Partner choice: 1 = Utilitarian, 0 = Deontological; 1) Own preference (i.e., participant's own preference): 1 = Utilitarian, 0 = Deontological.
